# Supplementary material for: Direct Laser Interference Ink Printing Using Copper Metal–Organic Decomposition Ink for Nanofabrication
Source: Nanomaterials (Basel). 2022 Jan 25;12(3):387. doi: 10.3390/nano12030387 (PMC8838969; doi:10.3390/nano12030387)
Supplement: Supplementary file 1 [file nanomaterials-12-00387-s001.zip › nanomaterials-1521102-supplementary.pdf]

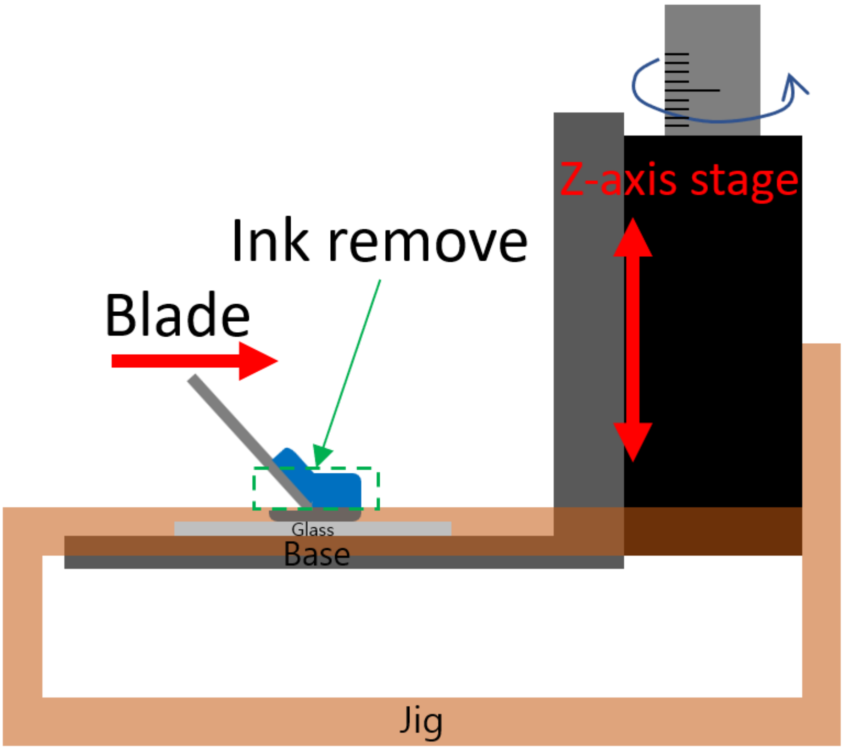

Figure S1. Blade coating process. overall coating procedure is as belows: Twenty microliters of the MOD ink is applied on the substrate. next, substrate is placed on the Z-axis stage base and placed proper height. then, gap between jig surface and glass substrate is few micrometers. After then, blade moves and remove the ink. because blade is located on jig surface, only few micrometrers thick ink remain.

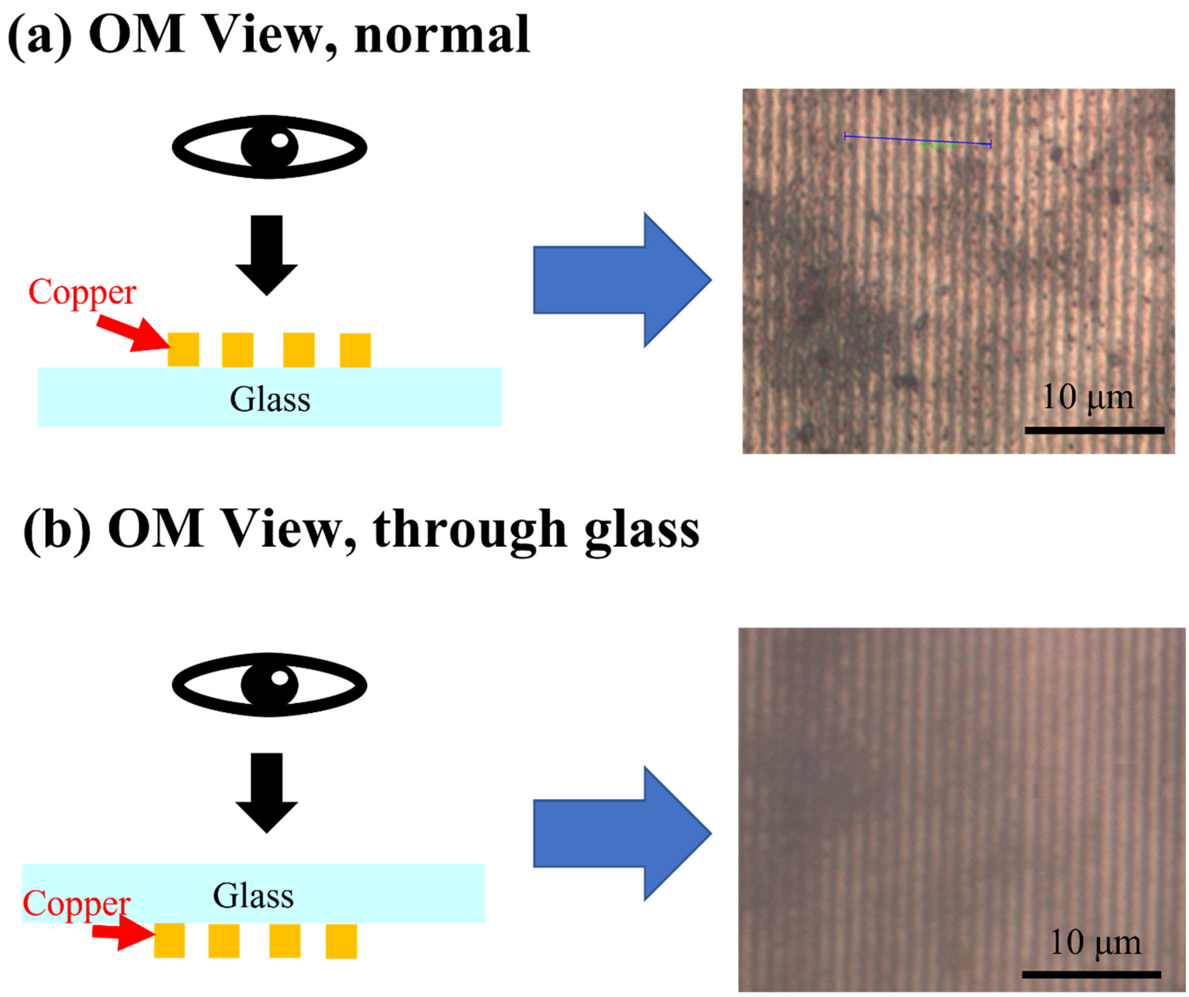

Figure S2. OM images of the copper electrode: (**a**) normal view and (**b**) view through the glass. These images show that the electrode consists of many copper lines arranged in parallel.

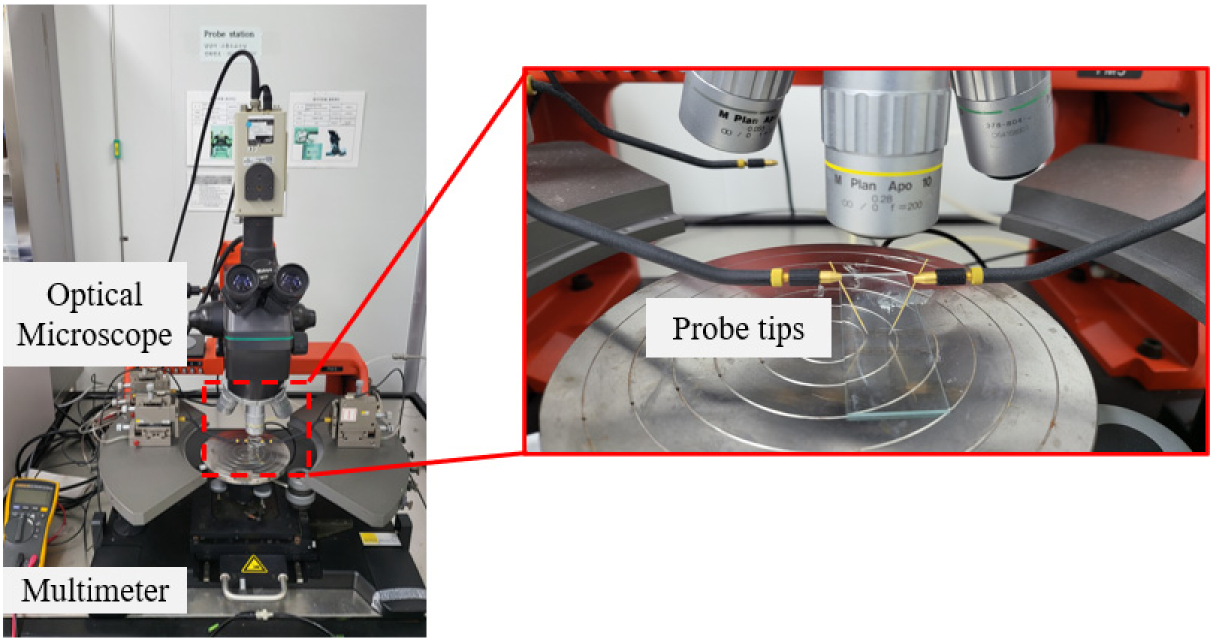

Figure S3. Setup for resistivity measurement.
